# Repeated Vertebral Column Resection (Re‐VCR) in Congenital Scoliosis With Curve Progression After Instrumentation Removal

**DOI:** 10.1111/os.70198

**Published:** 2025-11-06

**Authors:** Yinkun Li, Wanyou Liu, Benlong Shi, Zhen Liu, Saihu Mao, Jun Qiao, Zezhang Zhu, Yong Qiu

**Affiliations:** ^1^ Division of Spine Surgery, Department of Orthopedic Surgery, Nanjing Drum Tower Hospital The Clinical College of Nanjing Medical University Nanjing China; ^2^ Division of Spine Surgery, Department of Orthopedic Surgery, Nanjing Drum Tower Hospital The Affiliated Hospital of Nanjing University Medical School Nanjing China

**Keywords:** complications, congenital scoliosis, curve progression, instrumentation removal, revision surgery, vertebral column resection

## Abstract

**Objective:**

To evaluate the surgical efficacy of repeated vertebral column resection (Re‐VCR) after instrumentation removal in congenital scoliosis (CS) patients previously undergoing primary posterior spinal correction with VCR, and to analyze complications pertinent to revision surgery.

**Methods:**

In this retrospective cross‐sectional study, a total of 16 CS patients who underwent Re‐VCR following instrumentation removal between February 2013 and February 2022 were reviewed. Radiographic parameters were assessed pre‐ and post‐primary operation, pre‐removal, pre‐ and post‐revision and at the last follow‐up. Clinical data were also analyzed and recorded for each patient.

**Results:**

The indications for instrumentation removal were infection, implant failure, patient and family request, and persistent pain. The Cobb angle of the main curve, global kyphosis (GK), coronal balance (CB) and sagittal vertical axis (SVA) significantly progressed after instrumentation removal. The average progression rates of scoliosis and kyphosis were 5.3° ± 4.0°/year and 10.0° ± 7.2°/year. Following revision surgery, the Cobb angle of the main curve, GK, CB showed significant improvement (*t* = 10.694, *p* < 0.001; *Z* = −3.516, *p* < 0.001; *Z* = −2.664, *p* = 0.008). For Re‐VCR, the average extension of the fusion level was 2.9 ± 1.4 vertebrae proximally, 3.0 (2.0, 3.0) vertebrae distally and 5.4 ± 1.6 vertebrae in total. The average correction rates of the Cobb angle of the main curve and GK were 59.5% ± 23.4% and 53.7% ± 18.3% with no significant correction loss during follow‐up (*p* > 0.05). Compared with pre‐revision, the mean scores of pain, satisfaction, mental health and self‐image on the Scoliosis Research Society‐22 (SRS‐22) questionnaire improved at different levels. Intra‐revision complications included alert of neurophysiological monitoring and dural tear, while breakage of the distal L5 pedicle screw occurred in 1 (6.3%) patient 2 years after revision.

**Conclusions:**

Severe progression of deformity and trunk imbalance was frequently observed following instrumentation removal. The removal of instrumentation is not routinely recommended, and revision surgery employing Re‐VCR frequently necessitates an extension of the fusion level. Satisfactory radiographic and clinical outcomes following Re‐VCR were effectively maintained throughout the follow‐up period, but great caution should be exercised during Re‐VCR.

## Graphical abstract

The average extension of fusion level for repeated vertebral column resection (Re‐VCR) was 5.4 in total and the average correction rates of the Cobb angle of the main curve and kyphosis were 59.5% and 53.7%. Satisfactory radiographic and clinical outcomes after Re‐VCR could be achieved, but great care should be taken.

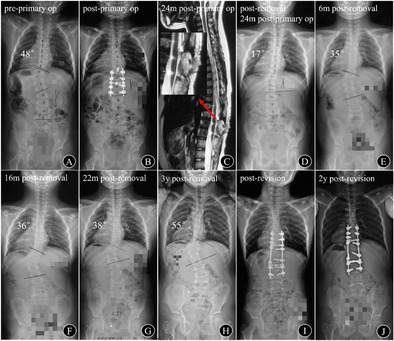

## 1 Introduction

Over recent decades, vertebral column resection (VCR) combined with short‐segment fusion has emerged as a recommended surgical approach for relatively young patients with congenital scoliosis (CS), aiming to preserve spinal longitudinal growth while minimizing excessive intervention [1, 2]. Zhuang et al. [2] documented significant improvement in coronal (63%) and sagittal (58%) trunk shift during over 2 years of follow‐up in 14 CS patients undergoing single‐stage posterior lumbosacral hemivertebra resection with short‐segment fusion. However, instrumentation removal is occasionally necessitated by implant‐related complications [3, 4, 5, 6, 7, 8, 9], including infection, persistent pain, implant failure, or protrusion. Critically, as the peak growth velocity in children remains unattained and anterior spinal flexibility persists despite fusion [10], curve progression frequently occurs following instrumentation removal. Wang et al. [10] reported approximate progression rates after instrumentation removal of 2.3°/year for scoliosis and 2.9°/year for kyphosis in CS patients. Consequently, revision surgery involving repeated vertebral column resection (Re‐VCR) is often required.

Previous research has demonstrated that satisfactory radiographic and clinical outcomes can be achieved following Re‐VCR [10, 11]. Oshima et al. [11] reviewed 55 patients who underwent Re‐VCR, reporting a mean correction rate of 48% for scoliosis and 52% for kyphosis. The mean number of VCR levels was significantly higher in the Re‐VCR group compared to the primary group (1.6 vs. 1.3, *p* = 0.005). Furthermore, the authors documented an intraoperative complication rate of 42% (23/55) and a postoperative complication rate of 24% (13/55), indicating that Re‐VCR entails greater technical challenges owing to anatomical distortions from prior surgeries. However, to the best of our knowledge, a paucity of relevant research exists on Re‐VCR in CS patients with curve progression following instrumentation removal.

Herein, based on a series of CS patients undergoing Re‐VCR for curve progression following instrumentation removal, this retrospective study was designed: (i) to evaluate the efficacy of Re‐VCR in CS patients after instrumentation removal; and (ii) to analyze the complications associated with Re‐VCR within this cohort.

## 2 Subjects and Methods

### 2.1 Patients

The present study was approved by the Hospital Clinical Research Ethics Committee (AF/SC‐08/03.0), and written informed consent was obtained from all participants or their legal guardians prior to enrollment. This retrospective study was conducted on CS patients undergoing posterior‐only spinal revision surgery at our institution between February 2013 and February 2022. Enrollment was limited to: (i) patients who underwent primary posterior spinal fusion with VCR followed by instrumentation removal during follow‐up; (ii) patients who underwent Re‐VCR due to curve progression; (iii) patients with more than 2‐year follow‐up after revision surgery; (iv) patients with intact radiographic and clinical data from primary surgery to the last follow‐up. The following exclusion criteria were applied: (i) patients who underwent combined anterior and posterior spinal fusion at primary surgery; (ii) patients who underwent partial instrumentation removal. Finally, a total of 16 patients (10 males and 6 females) meeting the inclusion/exclusion criteria were included, with an average age of 14.0 (11.3, 30.5) years.

### 2.2 Radiographic and Clinical Evaluations

**FIGURE 1:**
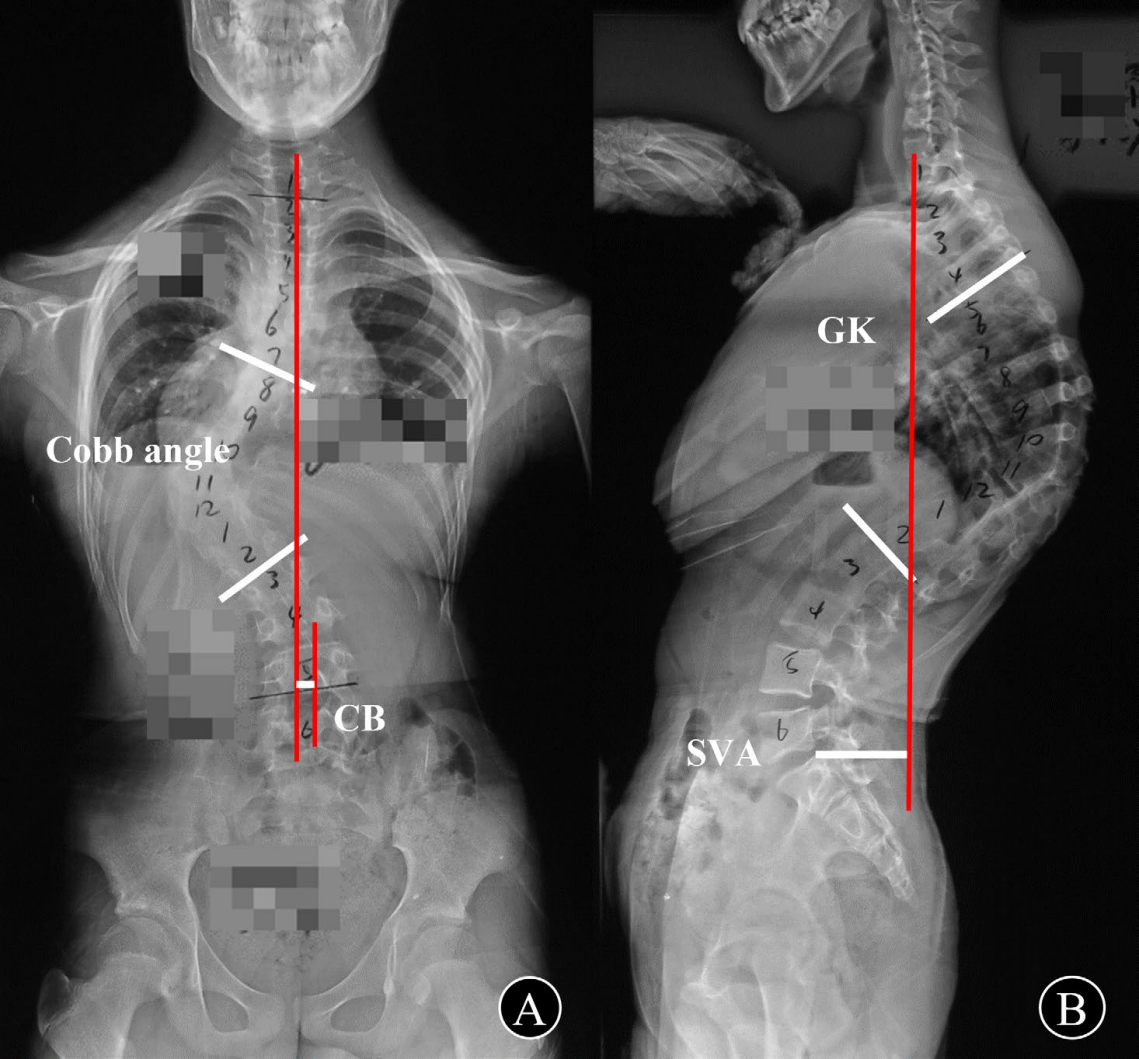
Illustration of the parameters measured on standing whole spinal *x*‐rays.

The following radiographic parameters (Figure 1) of pre‐ and immediate post‐primary operation, pre‐instrumentation removal, pre‐ and post‐revision, and the last follow‐up were measured on whole standing spinal *x*‐rays: (1) Cobb angle of the main curve, defined as the angle between the maximally tilted upper and lower end vertebrae in the coronal plane. (2) Global Kyphosis (GK), defined as the angle between the maximally tilted upper and lower end vertebrae in the sagittal plane. (3) Coronal Balance (CB), defined as the horizontal distance between the C7 plumb line and the central sacral vertical line. (4) Sagittal Vertical Axis (SVA), defined as the distance between the C7 plumb line and the posterior superior corner of S1.

Clinical data collected included the protocol of primary surgery, indications for instrumentation removal, reasons for revision surgery and the revision protocol. Time intervals were recorded from primary surgery to instrumentation removal and from instrumentation removal to revision surgery. Complications during revision surgery and follow‐up (e.g., infection, neurological deficit, implant failure, curve progression) were documented for each patient.

### 2.3 Clinical Assessment

Patients elder than 12 years were required to fulfill the Scoliosis Research Society–22 (SRS‐22) questionnaire both at pre‐revision surgery and at the last follow‐up in order to assess the improvement in quality of life.

### 2.4 Statistical Analysis

Data analysis was conducted using statistical software (SPSS 26.0, SPSS Inc., Chicago, IL). Continuous variables were tested for normality using the Shapiro–Wilk test. Normally distributed data are presented as mean ± standard deviation (^−^ *x* ± *s*) and compared using the paired Student's *t* test; non‐normally distributed data are presented as median (lower quartile, upper quartile) [M (Q1, Q3)] and compared using the Wilcoxon signed‐rank test. Comparisons of radiographic parameters were performed between pre‐ and immediate post‐primary surgery, pre‐instrumentation removal, pre‐ and post‐revision surgery, and last follow‐up. A *p* value < 0.05 was considered statistically significant.

## 3 Results

### 3.1 General Data

The mean age of patients at the time of primary surgery was 8.0 (5.0, 11.0) years. The average interval between the primary surgery and instrumentation removal was 24.0 (12.0, 33.0) months (7–60 months), including 1 (6.3%) within 1 year after the primary operation, 11 (68.8%) within 1–2 years, and 4 (25.0%) more than 2 years. The indications for instrumentation removal were categorized as follows: (i) infection in 12 cases (75.0%); (ii) implant failure in 2 cases (12.5%); (iii) patient and family request in 1 case (6.3%); (iv) persistent pain in 1 case (6.3%).

The average duration between instrumentation removal and revision surgery was 60.0 (37.5, 84.0) months (6–384 months), including 1 (6.3%) within 1 year after instrumentation removal, 1 (6.3%) within 1–2 years, and 14 (87.5%) more than 2 years. The indications for revision surgery were: (i) severe curve progression in all 16 cases (100%); (ii) trunk imbalance in 5 cases (31.3%); (iii) new neurological deficits secondary to kyphosis progression in 1 case (6.3%). Radiographic characteristics of spinal deformities were documented as follows: (i) kyphosis in 15 cases (93.8%), including angular kyphosis in 6 cases (37.5%); (ii) scoliosis in 14 cases (87.5%); (iii) hemivertebra growth in 8 cases (50.0%); (iv) severe vertebral rotation in 2 cases (12.5%). The mean operative duration for revision surgery was 357.5 (330.0, 445.0) minutes with an estimated blood loss of 1275.0 ± 427.0 mL.

### 3.2 Curve Progression After Instrumentation Removal

At pre‐primary operation, the mean values were 46.4° ± 29.7° for the Cobb angle of the main curve, 37.8° ± 10.8° for GK, 15.8 ± 9.9 mm for CB, and 19.3 ± 10.3 mm for SVA, respectively. At immediate post‐primary operation, significant corrections were observed in the Cobb angle of the main curve and GK, which were reduced to 22.3° ± 21.3° and 19.3° ± 8.6° (*t* = 5.175, *p* = 0.002; *t* = 7.620, *p* < 0.001). The CB and SVA were 9.6 ± 6.2 mm and 14.6 ± 5.8 mm respectively, showing a trend toward improvement (*t* = 1.935, *t* = 1.072, all *p* > 0.05). At pre‐instrumentation removal, no loss of correction was noted in any parameter (*t* = −1.916, *t* = −1.628, *t* = −0.272, *t* = 0.664, all *p* > 0.05). However, significant correction loss was evident in the Cobb angle of the main curve (66.6° ± 23.4°), GK (71.2° ± 17.6°), CB (16.5 (7.8, 23.6) mm) and SVA (18.0 (6.7, 49.1) mm) at pre‐revision (*t* = −3.772, *p* = 0.001; *t* = −6.440, *p* < 0.001; *Z* = −2.329, *p* = 0.020; *Z* = −2.120, *p* = 0.034, Table 1). The average progression rates of scoliosis and kyphosis were 5.3° ± 4.0°/year and 10.0° ± 7.2°/year, respectively Table 1.

**TABLE 1:** Comparison of radiographic parameters among pre‐primary operation, post‐primary operation, pre‐instrumentation removal, pre‐revision, post‐revision, and the last follow‐up.

Parameters	Cobb angle of main curve (°)	GK (°)	CB (mm)	SVA (mm)
Pre‐primary op	46.4 ± 29.7	37.8 ± 10.8	15.8 ± 9.9	19.3 ± 10.3
Post‐primary op	22.3 ± 21.3	19.3 ± 8.6	9.6 ± 6.2	14.6 ± 5.8
Pre‐removal	26.5 ± 23.6	23.8 ± 12.2	10.4 ± 9.2	13.2 ± 6.7
Pre‐revision	66.6 ± 23.4	71.2 ± 17.6	16.5 (7.8, 23.6)	18.0 (6.7, 49.1)
Post‐revision	29.7 ± 22.7	31.7 (27.0, 35.6)	8.4 (5.0, 18.2)	19.7 ± 12.3
Last follow‐up	30.1 ± 21.9	31.8 ± 15.9	6.8 (4.1, 17.5)	14.9 (9.3, 30.0)
Pre‐ versus post‐primary op	*t* = 5.175, *p* = 0.002	*t* = 7.620, *p* < 0.001	*t* = 1.935, *p* = 0.101	*t* = 1.072, *p* = 0.325
Post‐primary op versus pre‐removal	*t* = −1.916, *p* = 0.104	*t* = −1.628, *p* = 0.155	*t* = −0.272, *p* = 0.795	*t* = 0.664, *p* = 0.531
Pre‐removal versus pre‐revision	*t* = −3.772, *p* = 0.001	*t* = −6.440, *p* < 0.001	*Z* = −2.239, *p* = 0.020	*Z* = −2.120, *p* = 0.034
Pre‐ versus post‐revision	*t* = 10.694 *p* < 0.001	*Z* = −3.516, *p* < 0.001	*Z* = −2.664, *p* = 0.008	*Z* = −1.034, *p* = 0.301
Post‐revision versus last follow‐up	*t* = −0.524, *p* = 0.609	*Z* = −0.057, *p* = 0.955	*Z* = −0.994, *p* = 0.320	*Z* = 0.000, *p* = 1.000

Abbreviations: CB, coronal balance; GK, global kyphosis; SVA, sagittal vertical axis.

### 3.3 Surgery Strategies

Primary strategies included short segmental fusion with complete resection of hemivertebra in 9 cases and incomplete resection in 7 cases. Revision strategies for the 5 cases with trunk imbalance and the 16 cases with curve progression included extension of fusion levels to ensure the horizontalization of the upper and lower instrumented vertebrae, accompanied by massive bone graft. Fusion of compensatory curve was performed when the curve was structural. For patients with residual or incompletely resected hemivertebrae, thorough resection of the remaining hemivertebrae and adjacent discs was adopted. For the 1 case with new neurological deficits, spinal stability and decompression of the spinal cord were the priorities of revision surgery. Additionally, the satellite rod technique was employed if possible to achieve rigid and robust instrumentation.

### 3.4 Efficacy of Re‐VCR

**FIGURE 2:**
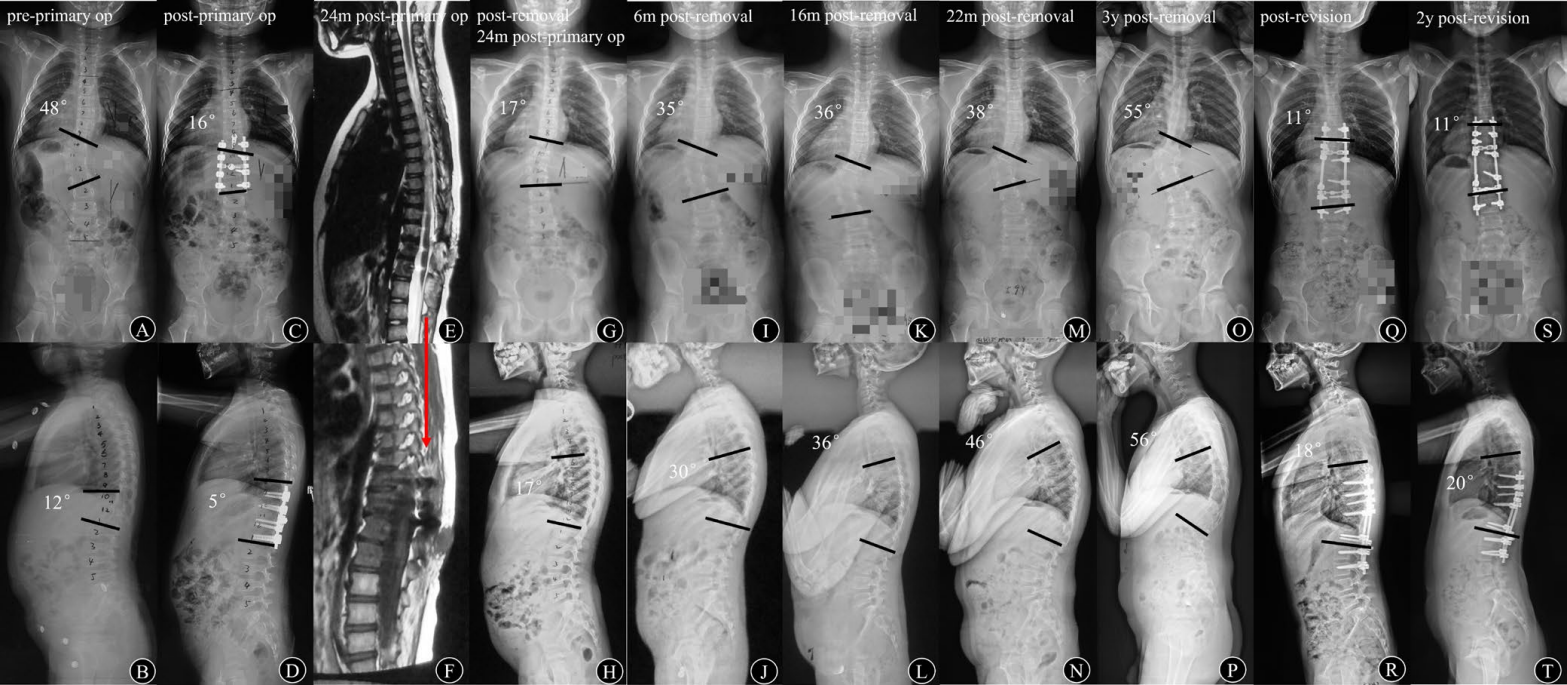
The patient was diagnosed as CS with *T*_11_ hemivertebra at 5 years old and the Cobb angle of the main curve and GK were 48° and 12°, respectively (A, B). Posterior hemivertebra resection and fusion were performed immediately (C, D). The instrumentations were removed at 24 months post‐primary operation due to late‐onset infection (E, F). At 3 years after instrumentation removal, the Cobb angle of the main curve rapidly progressed from 17° to 55° (G, I, K, M, O) and GK from 17° to 56° (H, J, L, N, P). Posterior revision surgery was performed and the Cobb angle of the main curve and GK were 11° and 18° immediately after revision (Q, R). No deterioration of deformity or trunk balance was observed during the 2‐year follow‐up (S, T).

**FIGURE 3:**
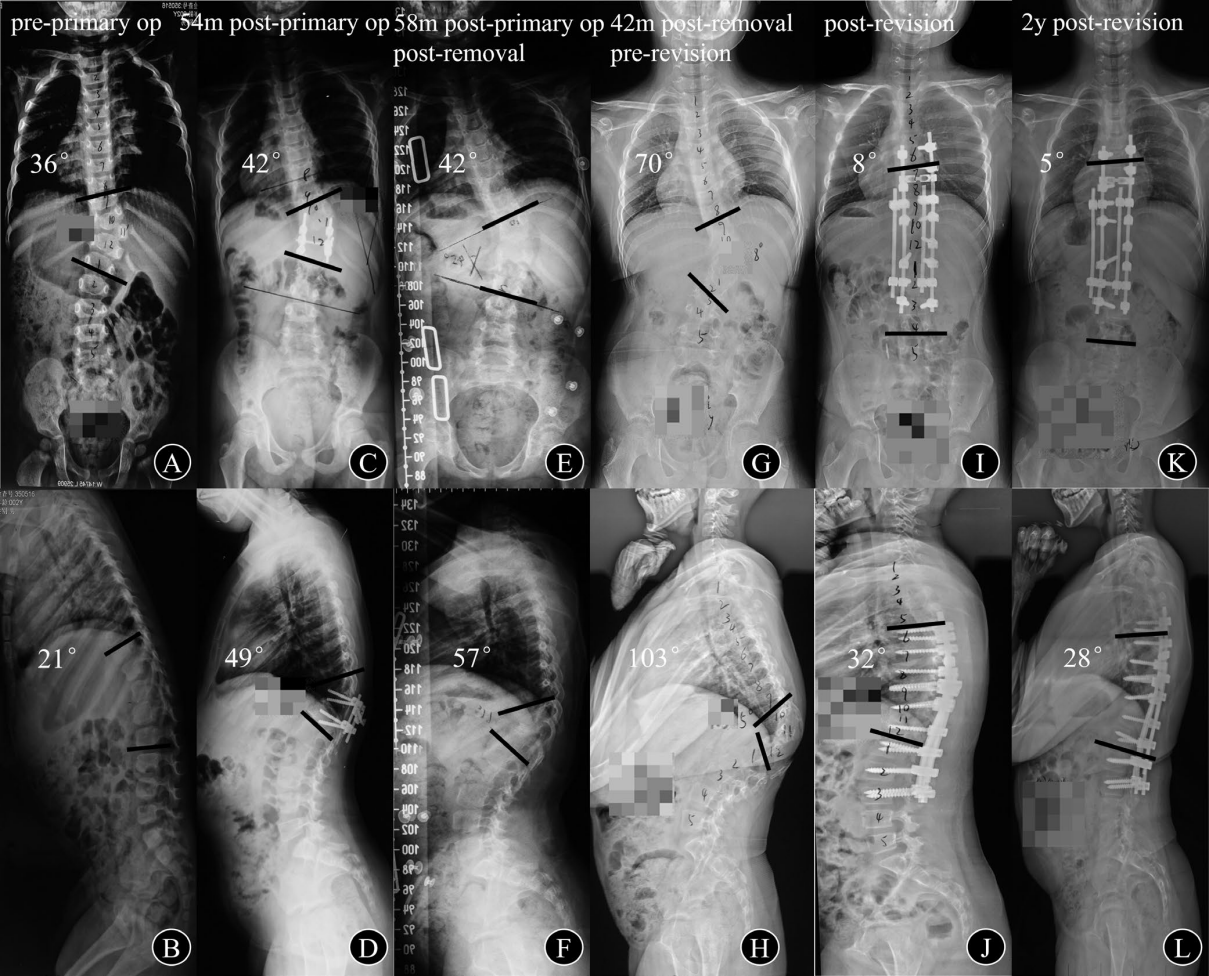
The patient was diagnosed with CS secondary to *T*_11_ hemivertebra at 3 years old, with the Cobb angle of the main curve and GK measuring 36° and 21°, respectively (A, B). Posterior hemivertebra resection and fusion were performed; however, the patient failed to attend regular postoperative follow‐up. Implant failure was observed 54 months postoperatively (C, D) and the instrumentations were removed (E, F). At 42 months after instrumentation removal, the Cobb angle of the main curve progressed from 42° to 70°, whereas the GK increased from 57° to 103° (E–H). Revision surgery involving thorough posterior resection of the residual hemivertebra, extension of fusion levels, and bilateral satellite rod instrumentation was performed. The Cobb angle of the main curve and GK were corrected to 8° and 32° immediately after revision (I, J). Radiographic outcomes remained stable with satisfactory maintenance of correction during the 2‐year follow‐up (K, L).

The mean extension of fusion level was 2.9 ± 1.4 vertebrae proximally, 3.0 (2.0, 3.0) vertebrae distally and 5.4 ± 1.6 vertebrae in total. At immediate post‐revision, the mean Cobb angle of the main curve was significantly corrected to 29.7° ± 22.7° with an average correction rate of 59.5% ± 23.4%, GK to 31.7° (27.0°, 35.6°) with an average correction rate of 53.7% ± 18.3% and CB to 8.4 (5.0, 18.2) mm (*t* = 10.694, *p* < 0.001; *Z* = −3.516, *p* < 0.001; *Z* = −2.664, *p* = 0.009), respectively. The mean follow‐up duration after revision was 26.5 (24.0, 32.3) months (24–60 months). At the last follow‐up, no significant correction loss was observed in any parameter (*t* = −0.524; *Z* = −0.057; *Z* = −0.994; *Z* = 0.000; all *p* > 0.05; Table 1). Two demo cases are displayed in Figures 2 and 3.

### 3.5 Quality of Life

A total of 12 patients responded to the SRS‐22 questionnaire at both pre‐revision and the last follow‐up. At pre‐revision, the mean scores of pain, satisfaction, mental health and self‐image were 2.5 ± 0.5, 3.4 (1.8, 3.7), 3.7 ± 0.2 and 3.2 ± 1.0, respectively. At the last follow‐up, these scores improved to 2.6 ± 0.5, 3.4 (2.0, 3.7), 3.8 (3.6, 4.0) and 3.6 ± 0.8, respectively, demonstrating improvements at different levels (*t* = −2.335, *p* = 0.040; *Z* = −2.000, *p* = 0.046; *Z* = −2.041, *p* = 0.041; *t* = −3.203, *p* = 0.008) Table 2.

**TABLE 2:** Comparison of the SRS‐22 domains between pre‐revision and the last follow‐up.

Domains	Pre‐revision	Last follow‐up	Pre‐ revision versus last follow‐up
Function	4.1 (3.3, 4.3)	4.1 (3.4, 4.3)	*Z* = −0.577, *p* = 0.564
Pain	2.5 ± 0.5	2.6 ± 0.5	*t* = −2.335, *p* = 0.040
Satisfaction	3.4 (1.8, 3.7)	3.4 (2.0, 3.7)	*Z* = −2.000, *p* = 0.046
Mental health	3.7 ± 0.2	3.8 (3.6, 4.0)	*Z* = −2.041, *p* = 0.041
Self‐image	3.2 ± 1.0	3.6 ± 0.8	*t* = −3.203, *p* = 0.008

### 3.6 Complications

Alert in intraoperative neurophysiological monitoring (IONM) was reported in 1 case (6.3%) during rod placement following Re‐VCR. The rods were removed and the IONM improved after further adequate decompression. Postoperative crus numbness was reported. Methylprednisolone and mannitol were administered for 3 days, resulting in complete recovery after 4 weeks of conservative treatment. A dural tear was found in 4 patients (25.0%) during Re‐VCR and intraoperative epidural suturing was performed. All patients recovered following postoperative bed rest and pressure dressing. Breakage of the distal L5 pedicle screw occurred in 1 patient (6.3%) 2 years after revision, which was considered to be related to the risk factors of lumbar mobility and stress. No correction loss was observed at the latest follow‐up and no second revision surgery was performed.

## 4 Discussion

Curve progression subsequent to instrumentation removal in adolescent idiopathic scoliosis (AIS) patients following corrective spinal surgery is well documented in the literature. Benes et al. [12] reported significantly greater mean major curve correction loss (9° vs. 1°, *p* < 0.001) and kyphosis progression (8° vs. 1°, *p* = 0.04) in the instrumentation removal cohort compared to the instrumentation exchange group. Moreover, the authors documented a revision rate of 7.7% (2/26). Potter et al. [13] observed an immediate correction loss of approximately 4° (range 0°–8°) after instrumentation removal, with continued settling of an additional 6° (10° total, *p* = 0.002) in the main thoracic curve in AIS patients. Furthermore, Farshad et al. [14] demonstrated a one‐third magnitude loss of correction following instrumentation removal during a 10‐year follow‐up period in an AIS cohort. According to previous studies [15, 16], several potential explanations such as pseudarthrosis, insufficient bony fusion and injury to paraspinal muscles could account for the coronal curve progression following instrumentation removal, often necessitating a three‐column osteotomy. However, curve progression following instrumentation removal in CS patients remains infrequently reported. Wang et al. [10] documented curve progression in CS patients, with scoliosis advancing from a mean of 9.84° to 16.42° (*p* = 0.017) and kyphosis increasing from 10.46° to 18.98° (*p* = 0.030) over a mean follow‐up of 43.9 months. In the present study, mean progression rates of 5.3°/year for scoliosis and 10.0°/year for kyphosis were observed during a mean follow‐up period of 60.0 months. Collectively, these studies indicate that patients undergoing instrumentation removal are at significant risk for rapid deformity progression during follow‐up. When implant removal is clinically unavoidable, consideration should be given to re‐instrumentation and revision surgery based on a comprehensive assessment of the patient's condition.

Previous studies [13, 15] have identified severe curve progression and trunk imbalance as the primary indications for revision surgery following instrumentation removal. Spontaneous compensation of shoulder balance may contribute to significant progression of both the primary and compensatory curves [15]. Furthermore, pseudarthrosis concealed by a thin bony shell within the fusion segment may also contribute to curve progression. According to Wang et al. [10], younger age, lower Risser's grade, and severe deformity represent potential risk factors for correction loss following instrumentation removal in CS patients. Notably, the authors reported that the primary operation involved the thoracolumbar segment in all patients requiring revision surgery, with the apex of curve progression consistently located at the crosslink area. Additionally, it was postulated that incomplete resection of the hemivertebra during the primary operation and residual growth plates in CS patients could precipitate rapid deformity progression and trunk imbalance after instrumentation removal. Consequently, Re‐VCR was deemed appropriate for this cohort and expected to yield substantial curve correction. In the present study, 8 patients with remaining or incompletely resected hemivertebrae underwent revision surgery due to trunk imbalance and severe curve progression. The application of unilateral or bilateral satellite rods during revision surgery serves to reinforce the fusion construct and mitigate the risk of implant failure [17, 18].

Luhmann et al. [19] demonstrated a reduction in the coronal Cobb angle from 58.6° to 26.3° among 41 AIS patients following revision surgery, achieving a correction rate of 55%. Shi et al. [20] documented satisfactory clinical and radiographic outcomes following revision surgery for CS patients with a single hemivertebra after a failed primary operation. In their cohort, the coronal Cobb angle, GK, CB, and SVA were effectively corrected from preoperative values of 40.2°, 42.8°, 23.6 mm, and 31.0 mm to postoperative values of 17.3°, 20.3°, 12.1 mm, and 15.7 mm, respectively. The average correction rates for the coronal Cobb angle and GK were 57.0% and 52.6%. In the present study, 16 patients underwent Re‐VCR subsequent to instrumentation removal. The average correction rates of the Cobb angle of the main curve and GK were 59.5% and 53.7%, respectively, demonstrating satisfactory radiographic and clinical outcomes without significant correction loss during follow‐up.

Complications associated with VCR remain a significant concern in spinal surgery. Iyer et al. [21] reported an overall complication rate ranging from 23.8% to 56.3% following VCR for rigid adult spinal deformity, with neurologic and pulmonary complications being predominant. Common pulmonary complications included pneumothorax and pleural effusions, whereas prevalent non‐neurologic complications encompassed fixation failures, compression fractures, proximal junctional kyphosis and pseudarthrosis. Lenke et al. [22] documented an overall complication rate of 58.5% (86/147) for severe pediatric spinal deformity. Their analysis further identified neurologic complications and estimated blood loss exceeding 2 L as the most frequent intraoperative complications, occurring in 46.3% (68/147) of cases. Additionally, Lenke et al. [23] reviewed 43 patients with severe spinal deformity who underwent VCR and found that the loss of neurogenic monitoring evoked potentials was most frequent in patients with kyphosis. In the present study, intraoperative neurophysiological monitoring alerts occurred in 1 patient and dural tears in 4 patients during osteotomy, indicating heightened surgical complexity and risk associated with Re‐VCR.

The limitations for this study were its relatively small sample size and retrospective character. Despite the small sample size, appropriate statistical methods such as parametric and non‐parametric tests were employed. Variations in decision‐making at primary surgery might have introduced certain biases into the results. A control group would enhance the conclusion of our study, while patients without revision surgery after instrumentation removal were not available in our center. Therefore, more longitudinal and multicenter studies with large sample sizes would be necessary to confirm the natural history after instrumentation removal. Despite the gratifying outcomes observed during the 26.5‐month follow‐up of the present study, more patients with longer follow‐up periods should be included for further comprehensive analysis.

## 5 Conclusions

The primary indications for instrumentation removal in CS patients who underwent posterior spinal correction surgery with VCR were infection and implant failure. Severe progression of deformity and trunk imbalance was often observed following instrumentation removal. The removal of instrumentation is not routinely recommended, and revision surgery employing Re‐VCR frequently necessitates an extension of the fusion level. Satisfactory radiographic and clinical outcomes following Re‐VCR were effectively maintained throughout the follow‐up period, but great caution should be exercised during Re‐VCR.

## Author Contributions

Y.L. and W.L. analyzed the statistics, wrote the main manuscript text and prepared Figure 2 and Tables 1 and 2. B.S., Z.L., S.M. and J.Q. designed the research, prepared Figure 1 and gave technical support. Z.Z. and Y.Q. designed the research and gave critical revision and technical support. All authors reviewed the manuscript.

## Disclosure

All authors listed meet the authorship criteria according to the latest guidelines of the International Committee of Medical Journal Editors. All authors are in agreement with the manuscript. Each author believes that the manuscript represents honest work and that the information is not provided in another form.

## Ethics Statement

This study was approved by the Institutional Ethics Committee (AF/SC‐08/03.0).

## Conflicts of Interest

The authors declare no conflicts of interest.

## Data Availability

The data that support the findings of this study are available on request from the corresponding author. The data are not publicly available due to privacy or ethical restrictions.
